# Microglia Play an Essential Role in Synapse Development and Neuron Maturation in Tissue-Engineered Neural Tissues

**DOI:** 10.3389/fnins.2020.586452

**Published:** 2020-11-19

**Authors:** Huimin Zhu, Xin Qiao, Wei Liu, Changyong Wang, Yuwei Zhao

**Affiliations:** Tissue Engineering Research Center, Academy of Military Medical Sciences and Department of Neural Engineering and BiologicalInterdisciplinary Studies, Institute of Military Cognition and Brain Sciences, Academy of Military Medical Sciences, Beijing, China

**Keywords:** 3D culture, microglia, neuron, reconstruction, tissue engineering, silk fibroin

## Abstract

In the process of constructing engineered neural tissues, we often use mixed primary neural cells, which contain microglia in the cell culture. However, the role that microglia play in the construction of engineered neural tissue has not been well studied. Here, we generated three-dimensional (3D) engineered neural tissues by silk fibroin/collagen composite scaffolds and primary mixed cortical cells. We depleted microglial cells by magnetic separation. Then, we analyzed the neural growth, development, mature and synapse-related gene, and protein expressions compared with the control engineered neural tissues with the microglia-depleted engineered neural tissues. We found that the engineered neural tissues constructed by magnetic separation to remove microglia showed a decrease in the number of synaptic proteins and mature neurons. These findings link microglia to neuron and synaptic maturation and suggest the importance of microglia in constructing engineered neural tissues *in vitro*.

## Introduction

Most of the *in vitro* approaches for constructing neuronal networks are based on two-dimensional (2D) cultures, which cannot recapitulate three-dimensional (3D) organizations, cell–cell interaction, or their network functions *in vivo* (Swistowski et al., 2010; Yang et al., 2016; Martin et al., 2018). *In vitro* methods to construct 3D neuronal networks that mimic both the structures and functions of neural tissues have been pursued by researchers in various fields, such as neural tissue engineering, neurodegenerative disease studies, and artificial intelligence. Several 3D *in vitro* research models in the form of cerebral organoids (Lancaster et al., 2013; Bagley et al., 2017; Birey et al., 2017), neurospheroids (Fennema et al., 2013; Jeong et al., 2015; van Pel et al., 2018), and hydrogel cultures (Irons et al., 2008; Ma et al., 2008; Koutsopoulos and Zhang, 2013; Sun et al., 2017) have been developed to study the specific processes of the brain that are challenging to investigate and manipulate *in vivo* in animal models or *ex vivo* in brain slices.

Silk protein has received recent attention for neural tissue engineering applications due to its excellent biocompatibility, controllable degradability, controllable mechanical properties, and the ability to be processed into multiple material forms (Kasoju and Bora, 2012; Kundu et al., 2013; Abbott et al., 2016; Huang et al., 2018). The unique silk protein properties protect the neurons from excitotoxicity and maintenance of adequate transfer because of biocompatibility and porous structure. The construction of the bioengineered neural tissues based on the silk fibroin (SF)–collagen composite scaffold can recapitulate functional neural networks (Tang-Schomer et al., 2014; Chwalek et al., 2015a,b).

The significant categories of cell sources for 3D neural cultures are pluripotent stem cells, neural stem cells, or primary cortical cells. Researchers often do not use a single type of cell for cell seeding, mainly because brain tissue is composed of different types of cells. *In vitro*, different types of brain cells play distinct roles in the process of neural cell remodeling and reassembly. Microglia are glial cells from the myeloid lineage. When brain tissue is damaged, microglia migrate to the site of damage and engulf the cellular debris. The recent discovery is that microglia also play some role in the uninjured brain. Microglia have shown that they can remove dead neurons as well as synapses (Schafer et al., 2012). New evidence suggests that microglia in the brain play an essential role in the synaptic organization, control of neuronal excitability, and trophic support during brain development (Stephan et al., 2012; Parkhurst et al., 2013; Grabert et al., 2016; Weinhard et al., 2018). However, in the tissue-engineered neural tissue model, the function of microglia, the neuron–microglia interactions are still unclear.

Here, we aimed to study the influence of microglia removal on neuronal survival, synapse development, and functional maturation of the neural network in engineered neural tissue constructed *in vitro* and tried to explain the interaction relationship between microglia and neurons in engineered neural tissue.

## Materials and Methods

### Preparation and Characterization of Silk Fibroin Porous Scaffolds

The silk solution was prepared as previously described (Rockwood et al., 2011). Briefly, silkworm cocoons were boiled in 0.02 M Na_2_CO_3_ for 30 min and washed with ddH_2_O to extract the sericin. The degummed silk was dissolved in 9.3 M Libr at 60°C for 4–6 h. The solution was dialyzed in ddH_2_O for 48 h and centrifuged at 12,000 rpm for 30 min to remove the aggregates. Then, 4 g NaCl (particle size ∼500 μm) was added to 2 ml of 8% SF solution in the containers for 48 h. The mixture was immersed in water for 48 h to extract the NaCl. Porous scaffolds were made as previously described (Altman et al., 2003). The SF scaffolds were sterilized and coated with poly-L-lysine before cell seeding.

The mechanical properties of the SF scaffolds were tested by an Instron mechanical tester (Instron 5900) and compared with rat and mouse brain tissues. All samples were applied 0.2 N load and compressed by the stress-relaxation tests, which was compressed stepwise at 5% of the height and relaxed for 500 s to establish equilibrium. The compressive load–compressive strain diagram was recorded. The compressive modulus was calculated as the minimum linear slope. All scaffolds and tissue samples were cut into 5 mm diameter and 2 mm height. Adult rat and mouse brain tissues were dissected from adult animals, stored in sterile PBS at 4°C, and tested within 4 h of animal euthanasia.

The SF scaffolds were dehydration treated, and then the morphology was analyzed by scanning electron microscopy (Hitachi S-3400N) at 5 kV. Samples were placed onto a copper plate, and gold sputtering was treated on the samples before observation.

### Isolation of Primary Cortical Cells

Isolation of primary cortical cells was assessed as previously described (Pacifici and Peruzzi, 2012). Briefly, primary rat cortical cells were isolated from embryonic day 16–18 SD rats. Cortical tissues were isolated, dissociated with trypsin (0.05%) followed with trypsin inhibition, centrifuged, and resuspended in NeuroBasal media with B-27 supplement and 2 mM L-glutamine.

### Magnetic Labeling and Separation of Microglial Cells

The isolated primary cortical cells were centrifuged and suspended in 80 μl buffer [0.5% bovine serum albumin (BSA) in phosphate buffered saline (PBS)] and 20 μl CD11b/c Microbeads (MACS, rat, 130-105-634) per 1 × 10^7^ cells for 15 min at 4°C and then resuspended in 500 μl buffer. LD column was placed in the magnetic field of a MACS Separator. The cell suspension was applied onto the column, and the flow-through unlabeled cells were collected, which were called microglia-depleted cortical cells. The magnetically labeled cells flushed out were microglia. The microglia were grown in Dulbecco’s Modified Eagle’s Medium (DMEM)–F12 plus 10% fetal bovine serum (FBS).

### Construct Assembly, Culture, and Evaluation

The concentrated cortical cells and microglia-depleted cortical cells were seeded on the SF scaffolds (5 mm diameter, 1 mm height) for 24 h, immersed with 200 μl collagen (3 mg/ml) for 30 min at 37°C, and adjusted pH to 7 by NaOH. The inside and edges of the scaffolds were immersed in collagen, and there were no bubbles in the added collagen. The construct seeded mixed cortical cells were called the control group, and the construct seeded microglia-depleted cortical cells were called the depleted group. For control cultures used for cell viability measurement and axon length assays, cells were seeded at 1 × 10^6^, 5 × 10^6^, and 1 × 10^7^ cells/composite scaffold. For control cultures and microglia-depleted group used for microtubule-associated protein 2 (MAP2) positive immunofluorescence staining, neural maturation, synapse development-related gene, and protein expressions based on SF/collagen scaffolds, cells were seeded at 5 × 10^6^ cells/composite scaffold.

#### Cell Viability Evaluation

A Live/Dead Kit (Invitrogen) was used to evaluate cell viability. Constructs were incubated with Calcein AM and EthD-1 at 37°C for 15–30 min. After incubation, cells were washed three times with PBS and imaged through a confocal microscope (Nikon A1). The images were taken from the surface to 10 μm deep at 2 μm intervals.

Cell Counting Kit-8 (CCK-8) assay (Dojindo) was used to assess the cell viability of 3D engineered tissue cultures and 2D cultures. CCK-8 was mixed in culture media (1:10) at different time points (day *in vitro* 1, 3, and 7 and one time every week up to 6 weeks) and incubated for 2 h at 37°C. Fluorescence was read at 450 nm on a microplate spectrophotometer (Molecular Devices). Three samples were used for each experiment.

#### Immunofluorescence Staining

After harvest, the samples were fixed with 4% paraformaldehyde for 30 min, washed with PBS, and treated with 0.3% Triton X-100 including 5% normal goat serum for 30 min, followed by incubation of primary antibodies overnight at 4°C. The next day after three 5 min washes, samples were incubated with secondary antibodies for 2 h at room temperature. Antibodies included anti-Tuj1, anti-CD11b/c, anti-IBA1, anti-MAP2, and goat anti-mouse or anti-rabbit secondary antibodies. Table 2 shows the information of the primary antibodies. Cell nuclei were stained with DAPI. Samples were again washed thoroughly with PBS before imaging. Fluorescence images were acquired by a Nikon A1 confocal microscope. The images were taken from the surface to 50–60 μm deep at 5 μm intervals.

#### Real-Time PCR Analysis

We assessed the expression level of genes (NCAM-L1, MAP2, Tuj1, NF-L, synaptophysin, GABAR1, etc.) associated with the neuron growth and microglial inflammatory response using real-time PCR. Briefly, total RNA was extracted with TRIzol reagent, and PCRs were carried out using a LightCycler 96 real-time quantitative thermal cycler (Roche) with SYBR Green PCR Mix (Toyobo). GAPDH was used as an internal reference. Table 1 shows all the primer sequences used in this study. All reactions were run in triplicate. The fluorescence intensity was recorded under the setting as follows: 20 s at 95°C and 30 s at 58°C for 40 cycles. Finally, the gene expression value of individual constructs was carefully calculated relative to *GAPDH* expression using the 2^–△△*Ct*^ method.

**TABLE 1:** Primer sequences in this study.

Genes	Forward primer	Reverse primer
Tuj1	GCCAAGTTCTGGGAGGCTCATC	GTAGTAGACACTGTAGCGTTCCA
GAP-43	AACGGAGACTGCAGAAAGCA	GCCTCGGGGTCTTCTTTACC
SNP-25	TGGATGAGCAAGGCGAACAA	TCCTGATTATTGCCCCAGGC
NCAM	CACCAGTGAGAGGGTGAGTG	CTCCAGTACATGGTGTCCTTT
NF-L	AATAAGTCGACGCTGCAGGACCTCAACCA	GATCTGAATTCCTGAGCCTGGTCTCTTC
MAP2	GAGAAGGAGGCCCAACACAA	TCTTCGAGGCTTCTTCCAGTG
IL-10	TTTAGGCGAGAAGCTGAAGG	TCTTCACAGGGCAGGAATCT
IL-1β	GACCTGTTCTTTGAGGCTGAC	TAGCCACGCCTTCTGTGACTCTAACT
TGF-β1	CCCGCATCCCAGGACCTCTCT	CGGGGGACTGGCGAGCCTTAG
IL-6	GACTGATGTTGTTGAGAGCCACTG	TAGCCACGCCTTCTGTGACTCTAACT
Synaptophysin	CATTCATGCGCGCACCTCCA	TTGCTGCCCATAGTCGCCCT
GABAR1	GTGCAAGTTAAATTGCGCTGCA	GCTTCCCAATATCCAATCTGCAGC
GAPDH	GATGGTGAAGGTCGGTGTGA	GGGATCTCGCTCCTGGAAG

**TABLE 2:** Information of antibodies used in this study.

Antibody	Type	Dilution	Source
Tuj1	Poly-rabbit	1:500	Abcam ab18207
CD11b/c	Poly-mouse	1:200	Abcam ab1211
MAP2	Poly-rabbit	1:500	Abcam ab32454
IBA1	Poly-mouse	1:200	Abcam ab15690
Synaptophysin	Poly-rabbit	1:200	Abcam ab32127

#### Western Blotting

Western blot analysis was carried out using control or microglia-depleted samples from day 7 and day 14 engineered tissues. The samples were treated in liquid nitrogen for 20 min, and then proteins extracted by Laemmli were homogenized on ice. The supernatant was collected after centrifugation at 12,000 rpm for 10 min. The concentration of proteins was detected by BCA Protein Assay Kit (Invitrogen). Equal amounts (60 μg) were separated on 12% SDS-PAGE gels and transferred to nitrocellulose membrane. The membranes were blocked with 5% defatted milk for 1 h, incubated with primary antibodies overnight at 4°C, and incubated with appropriate secondary antibodies for 1 h at room temperature. Table 2 shows the information of the primary antibodies. The labeled proteins were detected by enhanced chemiluminescence reagent (Applygen). GAPDH was used as the control to correct the band intensity.

### Statistical Analyses

All quantitative analyses were performed at least in triplicate, and the mean values were obtained. Results presented were based on the averages of data and standard error of the mean as error bars. The analysis used the Student’s *t*-test.

### Ethics Statement

All animal care and experimental protocols complied with the Animal Management Rule of the Ministry of Health, People’s Republic of China (Documentation No. 55, 2001). All procedures were approved by the Institutional Animal Care and Use Committee of the Academy of Military Medical Sciences, Beijing, China.

Table 3 shows all the abbreviations that appeared in this study.

**TABLE 3:** Abbreviations that appeared in this study.

Acronyms	Full acronym
NCAM	Neural cell adhesion molecule
GAP-43	Growth-associated protein-43
SNP-25	Synaptosomal-associated protein-25
SF	Silk fibroin
3D	Three-dimensional
2D	Two-dimensional
CNS	Central nervous system
MAP2	Microtubule-associated protein 2
BSA	Bovine serum albumin
GABA	Gamma aminobutyric acid

## Results

### Scaffold Characterization

**FIGURE 1:**
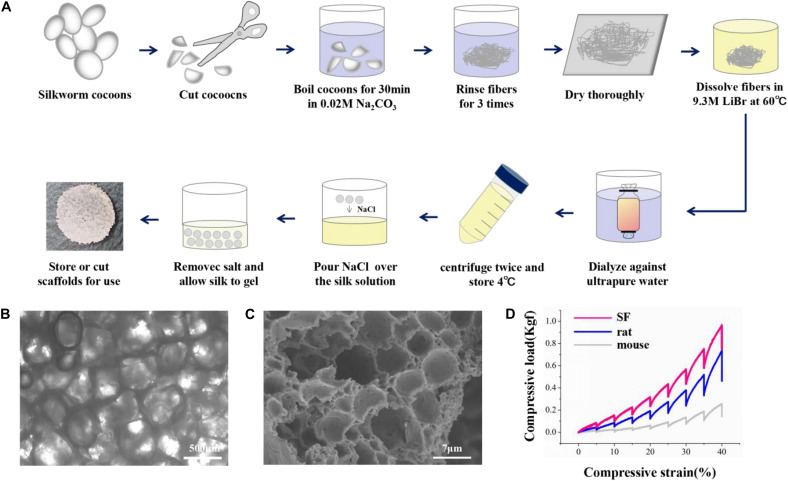
The preparation and characterization of the silk fibroin porous scaffolds. **(A)** Schematic from the silk fibroin extraction to the porous scaffolds. It takes about 4 days from the silk cocoons to the final silk fibroin porous sponges. **(B,C)** The bright-field microscope image and the scanning electron microscopy image show the inner structure of the SF sponges. **(D)** Compressive load-strain traces of the SF sponge, rat, and mouse cortical tissues.

The overall fabrication process from SF extraction to aqueous-based sponges is shown in Figure 1A. The pore size of the scaffolds was about <500 μm. The bright-field microscope image (Figure 1B) and SEM image (Figure 1C) showed the high porosity of the scaffolds, which allowed sufficient exchange of nutrients, oxygen, and wastes. The mechanical stiffness of the scaffolds and the cortical tissues of mouse and rat were tested by confined compression tests. The respective load-strain trace is shown in Figure 1D. The SF scaffolds had a modulus of 32.2 ± 9.7 kPa higher than a rat brain of 12.1 ± 2 kPa and a mouse brain of 3.1 ± 0.1 kPa.

### Biofabrication of 3D Engineered Neural Tissues

**FIGURE 2:**
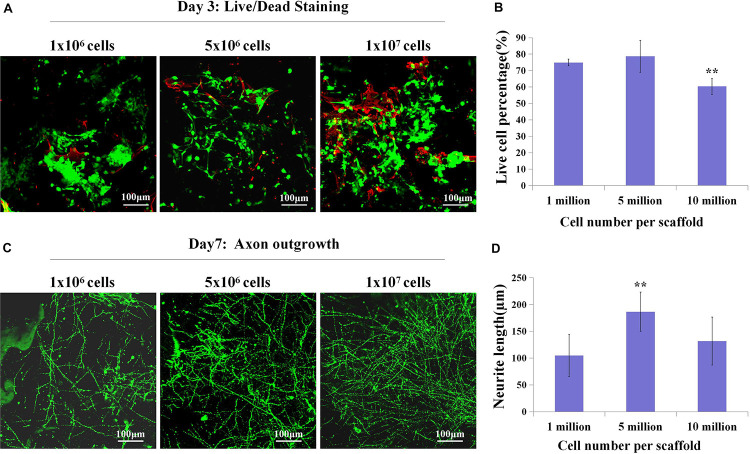
The live cell percentage and axon length with different cell densities. **(A,B)** Live (green)/Dead (red) staining showed live cell percentage with different cell densities on the SF sponges on day 3. **(C,D)** Immunochemistry with anti-Tuj1 indicated the neuronal axon length of different cell densities at day 7. **p < 0.01.

Since we have fabricated the SF sponges, the isolated primary cortical cells were cultured on the SF sponge. After the cortical cells were attached to the SF sponges, the scaffolds were filled with the collagen matrix to allow axon outgrowth and network formation. The 3D constructs viability of different cell seeding densities at 3 days of culture were stained with Live/Dead to evaluate the cell viability (Figures 2A,B). Confocal images showed that the dead cells percentage gradually increased as the cell seeding density increased. The 3D constructs growth of different cell seeding densities after 7 days of culture were immunostained with anti-Tuj1 fluorescent. The results showed that the protein expression of Tuj1 protein of different cell seeding densities was dissimilar. As cell seeding density increases, the more complex the neural network is formed morphologically (Figures 2C,D). The engineered neural tissue viability and axon outgrowth were found to be affected by cell density. Figure 2 shows that the most suitable cell seeding density was 5 × 10^6^ cells/scaffold, which showed the prolonged axon and higher proportion of live cells.

### 3D Engineered Neural Tissues Growth

**FIGURE 3:**
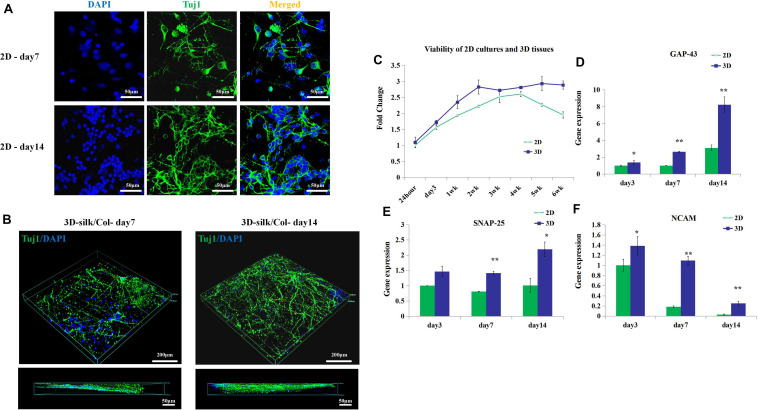
3D engineered neural tissue growth compared with 2D cultures. **(A)** Fluorescence images of 2D cultures on day 7 and day 14. **(B)** Fluorescence images of 3D fluorescence images of confocal z stacks of day 7 and day 14 neuronal axon on the SF sponges immunostained with Tuj1 in green and DAPI in blue. **(C)** Viability of 2D cultures and 3D engineered neural tissue cultures at 24 h, 3, 7 days, and 2–6 weeks assayed with CCK-8 and expressed relative to 24-h levels. **(D–F)** Expression of growth-associated protein 43 (GAP-43), neural cell adhesion molecule L1 (NCAM-L1), and synaptosomal-associated protein 25 (SNP-25) mRNA in 2D culture (green) and 3D engineered neural tissues culture (deep blue) at day 7 and day 14 relative to day 3 expression. *p < 0.05 and **p < 0.01.

Further, we observed the cells viability, axon growth of the engineered neural tissues *in vitro* compared with 2D culture. By confocal images, we observed that the axon growth of the engineered neural tissues was opposite from the 2D cultures (Figures 3A,B). The nucleus was anchored on the surface of porous sponges, and the axons penetrated the gel along with culture time. 3D engineered neural tissue culture showed a more prolonged axon than 2D culture.

The viability of 3D engineered neural tissues and 2D cultures was assayed by the CCK-8 at 24 h, 3, 7 days, 2, 3, 4, 5, and 6 weeks. 3D engineered neural tissue culture showed higher viability by OD value (Figure 3C). After 4 weeks, cortical cells viability in 2D cultures showed a decrease, and cortical cells in 3D cultures still maintained high viability. This result demonstrated that the SF scaffold would promote long-term cell survival compared with 2D cultures. Gene expression also showed the differences between 2D cultures and 3D cultures.

After 3 days of culture, the expression of growth-associated protein 43 (GAP-43) and NCAM-L1 was observed to be significantly upregulated for 3D cultures grown on SF/collagen composite materials compared with 2D cultures (1.4-fold). Similarly, after 7 days of culture, the expression of GAP-43, SNAP-25, and NCAM-L1 was observed to be significantly upregulated for 3D cultures compared with 2D cultures (2.7-, 1.8-, and 6-fold, respectively). The calculated expression levels of GAP-43, SNAP-25, and NCAM-L1 at 14 days of culture amounted to 2.7-, 2.2-, and 8.8-fold for 3D cultures compared with 2D cultures (Figures 3D–F). 3D engineered neural tissues showed higher expression levels of regenerative growth (GAP-43), synaptogenesis (SNAP-25), and neuronal adhesion (NCAM-L1) compared with 2D cultures at day 3, day 7, and day 14.

### Depletion of Primary Microglia by Magnetic Separation

**FIGURE 4:**
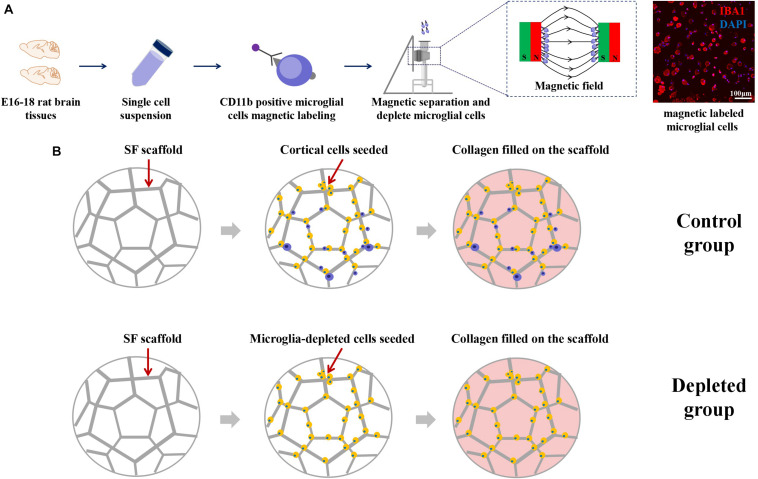
**(A)** Schematic from the isolation of cortical cells to the separation of microglial cells. Immunofluorescence images showed that the large proportion of the magnetically separated cells was IBA1 positive cells. **(B)** The isolated primary cortical cells and microglia-depleted cortical cells were cultured on the SF sponge, respectively. After cells were attached to the SF sponges, the scaffolds were filled with the collagen matrix to allow axon outgrowth and network formation.

Schematic images of magnetic separation of primary microglia are shown in Figure 4A. Immunofluorescence studies were performed to evaluate the depletion of the microglia after magnetic separation. The magnetically labeled cells flushed out showed IBA1 positive, indicating that the labeled cells were microglia, and most of the microglia were depleted by magnetic separation. We called the engineered neural tissues containing microglia the control group and the engineered neural tissues depleting microglia the depleted group (Figure 4B).

### The Maturation of Neurons and Synapse Development Were Decreased in the Microglia-Depleted 3D Tissues

**FIGURE 5:**
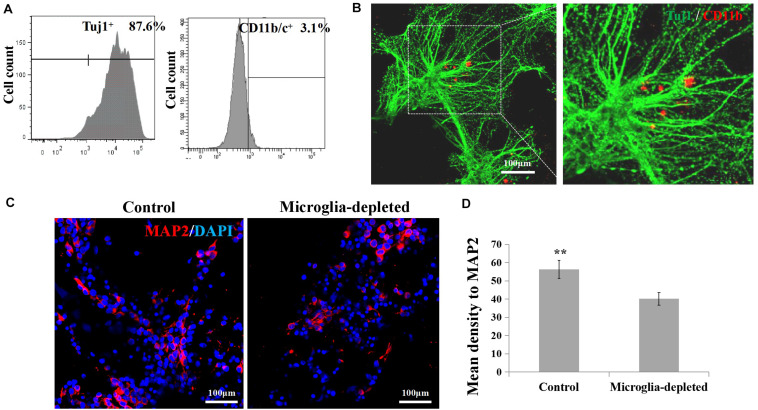
**(A,B)** Proportion and morphological distribution of neurons and microglia of the primary cortical cells. **(C)** Fluorescence images of day 7 axons immunostained with MAP2 in red and DAPI in blue. **(D)** Expression of microglia-related pro-inflammatory factor (IL-1β, IL-6) and inflammatory factor (IL-10, TGF-β) in the 3D engineered neural tissues (control group) and 3D engineered microglia-depleted neural tissues (depleted group). **p < 0.01.

By flow cytometry (Figure 5A), the proportion of the neurons in the isolated primary cortical cells is about 87.6 ± 5.2% (*n* = 3); the proportion of microglia is about 3.1 ± 0.25% (*n* = 3). To visualize microglia and neuron in the engineered neural tissues, we labeled microglia with CD11b/c, a neuron with Tuj1 using immunofluorescence staining. The Tuj1 and CD11b/c double-stained results of the control group revealed that in some cases, microglia were surrounded by neuron cell body and axon (Figure 5B). After 7 days of culture, the expression levels of MAP2 of the depleted group were lower than those of the control group (Figures 5C,D).

**FIGURE 6:**
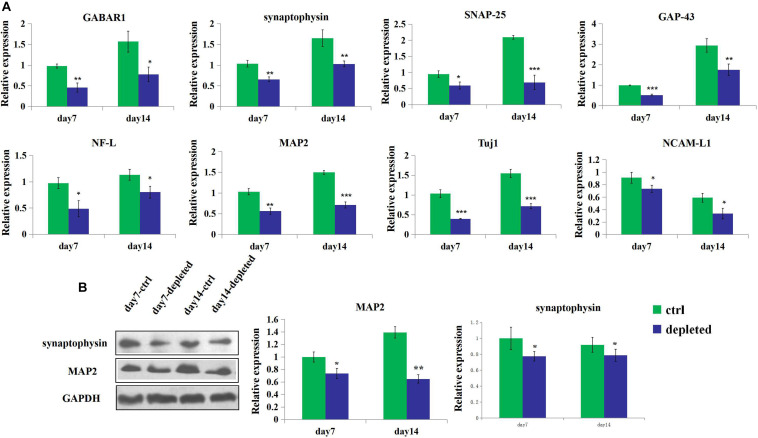
Gene and protein expression of the 3D engineered neural tissues. **(A)**. Expression of GABAR1, synaptophysin, SNAP-25, GAP-43, NCAM-L1, NF-L, MAP2, and Tuj1 mRNA in the control group (light blue) and the microglia-depleted group (deep blue) at day 14 relative to the day 7 expression. **(B)** Expression of synaptophysin, MAP2, and GAPDH protein in the control group (green) and the microglia-depleted group (deep blue) at day 14 relative to the day 7 expression. *p < 0.05, **p < 0.01, and ***p < 0.001.

3D neural tissues (control group) tended to have higher expression level of the neuron (Tuj1), mature neuron (MAP2), neuronal adhesion molecular (NCAM-L1), synaptogenesis (synaptophysin), neurofilament (NF-L), and receptors for gamma-aminobutyric acid (GABAR1) than the microglia-depleted 3D neural tissues (depleted group) as shown in Figure 6A. Especially after 4 days of culture, the expression of GABAR1 and Tuj1 was significantly upregulated for the control group compared with the microglia-depleted group (2.9- and 2.5-fold, respectively). The expression of NF-L after 7 days and GABAR1 after 14 days was significantly upregulated for the control group compared with the microglia-depleted group (4.3- and 2.4-fold, respectively). The expression levels of MAP2 and synaptophysin of the depleted group were lower than those of the control group at day 7 and day 14, respectively. These results of gene expression were consistent with the results of protein expression by Western blot (Figure 6B), suggesting that microglia play an essential role in synapse development and neuron maturation in tissue-engineered neural tissues.

In summary, we generated 3D engineered neural tissues based on the SF–collagen scaffolds. We compared the viability, the axon outgrowth length, and the expression from different cell densities. On this basis, we depleted microglia by magnetic separation and seeded the cortical cells, which remove microglia on the SF–collagen composite scaffold. The effect of the microglia depletion on the structure, gene, and protein expression was determined by immunocytochemical method, mRNA gene expression, and Western blot. We demonstrated that the maturation of neurons and synapse development were decreased in the microglia-depleted 3D tissues group compared with the control group. It is proven that microglia play an essential role in synapse development and neuron maturation in tissue-engineered neural tissues.

## Discussion

In this study, we mainly studied the role of microglia in the construction of 3D tissue-engineered neural tissues. We found that the depletion of microglia affects the synapse development and neuron maturation in the 3D neural tissues. This indicated that it is best to use mixed cortical cells of cells differentiated from neural progenitor/stem cells as seeded cells in the construction of engineered neural tissues, which is beneficial to constructing the functional engineered organization.

Microglia are homologous macrophage cells that, in addition to providing surveillance and clearance role, can engulf synapses in uninjured brains and achieve synaptic pruning during postnatal development (Prinz and Priller, 2014; Dong et al., 2018; Jung and Chung, 2018; Surinkaew et al., 2018). Paolicelli et al. demonstrated that microglia are involved in the development of brain wiring in newborn mice, and that disrupting microglia–synapse interactions delays the maturation of synaptic circuits (Paolicelli et al., 2011; Norris et al., 2018).

Engineered neural tissue construction is a hot topic in recent years. In recent years, researchers studied microglia-related inflammation or fractal analysis in tissue engineering (Jiang et al., 2016; Koss et al., 2017). However, until now, the role of microglia in the engineered neural tissue construction *in vitro* has not been clarified. Clarifying the role of microglia in the 3D engineered neural tissues contributes significantly to the engineered tissue construction and 3D models in neuroscience.

Obtaining or isolating microglia *in vitro* is different because of the cellular heterogeneity. Researchers usually use the method that is divided into three sections: mixed microglial, astroglial, and oligodendroglial cell culture; culture maintaining; and isolation of other cells except for microglia by shaking or digestion method (Ni and Aschner, 2010; Tamashiro et al., 2012; Roy, 2018). Here, we used a magnetic separation for isolation of primary microglia from primary mixed cortical cells. This method can highly and efficiently remove pure microglia from the mixed cortical cells by magnetic separation. The removed microglia obtained by this method were fully functional and morphologically like microglia obtained by conventional isolation methods.

In summary, microglia play a critical role in synapse development and neuron maturation in tissue-engineered neural tissues. Based on our findings, the maturation of neurons and synapse development were decreased in the microglia-depleted 3D bioengineered neural tissues. Microglia affect engineered neural tissues neuron maturation and synapse development probably through three ways: (1) microglia can remove dead and dying neurons and further affect the viability and remodeling of the engineered neural tissues; (2) in addition to some pro-inflammatory and inflammatory factors, microglia also secrete some neurotrophic factors; and (3) microglia remodel, run, or engulf synapse. However, further research is needed to illuminate the mechanisms on how the microglia affect neuron maturation and synapse development and related signal pathway.

## Data Availability Statement

The original contributions presented in this study are included in the article, further inquiries can be directed to the corresponding author.

## Ethics Statement

The animal study was reviewed and approved by the Institutional Animal Care and Use Committee of Academy of Military Medical Sciences.

## Author Contributions

HZ, XQ, and YZ designed the project. HZ wrote the manuscript and performed the experiments. HZ and XQ analyzed the data. CW supervised the project. All authors contributed to the article and approved the submitted version.

## Conflict of Interest

The authors declare that the research was conducted in the absence of any commercial or financial relationships that could be construed as a potential conflict of interest.
